# Supplementary material for: Multi-omic enriched blood-derived digital signatures reveal mechanistic and confounding disease clusters for differential diagnosis
Source: Front Netw Physiol. 2026 Jul 29;6:1747187. doi: 10.3389/fnetp.2026.1747187 (PMC13461528; doi:10.3389/fnetp.2026.1747187)
Supplement: Supplementary file 1 [file Supplementaryfile1.docx]

# **Supplementary Material**

Table S1: Disease profiles grouped by hierarchical clustering (cut at distance = 0.02). Profile IDs are omitted for clarity; only disease names are shown.

| **Cluster** | **Number of Diseases** | **Disease Names** |
| --- | --- | --- |
| 1 | 1 | Acute Myeloid Leukemia (AML) |
| 2 | 1 | Pancytopenia |
| 3 | 1 | AML2 |
| 4 | 1 | Lymphoma2 |
| 5 | 2 | Viral Lower Respiratory Tract Infection; Bacterial Pneumonia |
| 6 | 6 | Myeloma; Acquired Lymphopenia; CKD v2; Aortic Dissection; Meningitis v2; Febrile Neutropenia |
| 7 | 1 | Myelodysplastic Syndrome |
| 8 | 1 | Pancytopenia v2 |
| 9 | 80 | Microcytic Anaemia; Microcytic Anaemia v2; Normocytic Anaemia; Mixed Micro/Macrocytic Anaemia; Polycythaemia; Essential Thrombocythaemia; Acute Haemolytic Anaemia; Myelofibrosis; Drug-induced Neutropenia; Macrocytic Anaemia; Chronic Myeloid Leukemia; Chronic Lymphocytic Leukemia; Acute Blood Loss (Upper GI); Acute Blood Loss (Lower GI); Chronic Blood Loss; Allergic Reaction; Acute IBD Flare; Type 1 Diabetes; Type 2 Diabetes; Diabetic Ketoacidosis; Hyperthyroidism; Hypothyroidism; Hyper-/Hypokalaemia; Hyper-/Hypocalcaemia; Acute Kidney Injury (v1–v3); Chronic Kidney Disease; Hyponatraemia (Hypo-/Hyper-/Normovolaemic); Hypernatraemia; Adrenal Insufficiency; Hypophosphataemia; Hypomagnesaemia; Myopericarditis; Pericarditis; Infective Endocarditis; Myocardial Infarction (NSTEMI/STEMI); Heart Failure; Pulmonary Embolism; Deep Vein Thrombosis; Urinary Tract Infection; Pyelonephritis; Bacterial Pharyngitis; COPD; Asthma; Bronchiectasis; Empyema; Gastroenteritis; Hepatitis (v1/v2); Gilbert Syndrome; Cholangitis; Biliary Colic; Cellulitis; Osteomyelitis; Meningitis; Cholangiopathy; Atypical Pneumonia; Acute Pancreatitis; Chronic Diarrhoea (Coeliac); Diverticulitis; Gout; Giant Cell Arteritis; Rheumatoid Arthritis; Eosinophilic Vasculitis; Polymyalgia Rheumatica |
| 10 | 3 | CML2; CLL2; Lymphoma |
| 11 | 1 | ALL2 |
| 12 | 1 | Heparin-Induced Thrombocytopenia (HIT) |
| 13 | 1 | Immune Thrombocytopenia (ITP) |
| 14 | 1 | Acute Lymphoblastic Leukemia (ALL) |
| 15 | 1 | Thrombotic Thrombocytopenic Purpura (TTP) |
| 16 | 1 | Chronic Liver Disease |

| **Contrast** | **Feature** | **Hedges’ g** | **p-value** | **q-value (BH)** |
| --- | --- | --- | --- | --- |
| Infection vs Rest | MCV (fL)_t1 | -0.586 | 6.82E-07 | 3.70E-05 |
| Infection vs Rest | MCV (fL)_t3 | -0.632 | 7.98E-06 | 2.19E-04 |
| Rheumatological vs Rest | Segmented neutrophils (1000/µL)_t3 | -0.541 | 1.07E-05 | 5.89E-04 |
| Infection vs Rest | MCV (fL)_t5 | -0.488 | 3.77E-04 | 6.91E-03 |
| Rheumatological vs Rest | WBC (1000/µL)_t3 | -0.511 | 2.82E-04 | 7.75E-03 |
| Infection vs Rest | WBC (1000/µL)_t2 | 0.513 | 1.31E-03 | 1.80E-02 |
| Haematological vs Rest | Segmented neutrophils (1000/µL)_t5 | -0.735 | 1.34E-04 | 2.26E-02 |
| Haematological vs Rest | Segmented neutrophils (1000/µL)_t4 | -0.727 | 7.44E-04 | 2.26E-02 |
| Rheumatological vs Rest | WBC (1000/µL)_t2 | -0.387 | 1.35E-03 | 2.47E-02 |
| Rheumatological vs Rest | Segmented neutrophils (1000/µL)_t2 | -0.46 | 2.08E-03 | 2.86E-02 |
| Infection vs Rest | WBC (1000/µL)_t3 | 0.715 | 3.78E-03 | 2.97E-02 |
| Infection vs Rest | MCV (fL)_t4 | -0.511 | 3.76E-03 | 2.97E-02 |
| Infection vs Rest | WBC (1000/µL)_t1 | 0.389 | 3.39E-03 | 2.97E-02 |
| Infection vs Rest | WBC (1000/µL)_t4 | 0.705 | 4.53E-03 | 3.12E-02 |
| Infection vs Rest | WBC (1000/µL)_t5 | 0.823 | 6.01E-03 | 3.30E-02 |
| Infection vs Rest | MCV (fL)_t2 | -0.501 | 5.64E-03 | 3.30E-02 |
| Haematological vs Rest | RBC (million/µL)_t4 | -0.844 | 2.05E-05 | 4.71E-02 |

**Table S2. Top 20 time-series feature-level effect sizes across disease-system contrasts**

Feature-level differences between disease systems and the remainder of the dataset were quantified using Hedges’ g across longitudinal time-series measurements (t1 to t5). Statistical significance was assessed using Welch’s t-test or Mann–Whitney U test depending on normality (Shapiro–Wilk test), with Benjamini–Hochberg correction applied for multiple comparisons. Features are ranked by adjusted q-values and effect size magnitude. Dominant signals arise from red blood cells (RBC), neutrophils, mean corpuscular volume (MCV), and white blood cell count (WBC), demonstrating consistent discriminative patterns across timepoints.

SVHC hierarchical clustering confirmed that the time-series global disease-profile structure is statistically robust, identifying 25 validated clusters (p < 0.01) corresponding to statistically robust intra-cluster correlation blocks. Complementary effect size analysis reveals that these clusters are driven by coherent, system-specific hematologic trajectories over time, moderate-to-large, system-specific hematologic separations (Hedges’ g up to ~0.8, q < 0.05 after correction), with consistent signals across longitudinal timepoints.

Importantly, the recurrence of the same features (e.g., MCV, WBC, neutrophils) across multiple timepoints (t1–t5) indicates that disease differentiation is not static but reflects stable longitudinal dynamics, supporting a time-resolved, modular organization of the digital blood phenotypic space. Infection profiles exhibit consistent leukocytic elevation patterns, rheumatologic conditions show coordinated suppression or redistribution of immune cell populations, and hematological diseases demonstrate the largest magnitude deviations in erythroid and myeloid compartments. These findings support a complex system, temporally structured (i.e., attractor-like) architecture in which disease states are defined by reproducible trajectories of hematological patterns.

| **system_a** | **system_b** | **pearson_r** | **pearson_p** | **spearman_rho** | **spearman_p** |
| --- | --- | --- | --- | --- | --- |
| Other | Cardiac | 0.999729 | 2.98E-88 | 0.986265 | 3.93E-43 |
| Other | Other | 0.99961 | 4.84E-84 | 0.989242 | 6.28E-46 |
| Haematological | Infection | 0.999597 | 1.12E-83 | 0.993759 | 3.60E-52 |
| Other | GI_Hepatic | 0.99952 | 1.19E-81 | 0.985727 | 1.08E-42 |
| Haematological | Other | 0.999457 | 3.07E-80 | 0.970158 | 2.74E-34 |
| Other | Other | 0.999442 | 6.17E-80 | 0.984958 | 4.30E-42 |
| Other | Other | 0.999407 | 3.12E-79 | 0.977414 | 1.86E-37 |
| Other | Cardiac | 0.99936 | 2.42E-78 | 0.97652 | 5.16E-37 |
| Haematological | Other | 0.99935 | 3.63E-78 | 0.960414 | 4.33E-31 |
| Other | Other | 0.999347 | 3.98E-78 | 0.981817 | 6.29E-40 |
| Other | Other | 0.999318 | 1.28E-77 | 0.983634 | 3.95E-41 |
| Cardiac | GI_Hepatic | 0.999312 | 1.64E-77 | 0.977081 | 2.74E-37 |
| Haematological | Infection | 0.99922 | 4.43E-76 | 0.967898 | 1.84E-33 |
| Other | Other | 0.999218 | 4.77E-76 | 0.946801 | 9.21E-28 |
| Other | Cardiac | 0.999215 | 5.39E-76 | 0.988943 | 1.30E-45 |
| Haematological | Other | 0.999209 | 6.57E-76 | 0.968878 | 8.21E-34 |
| Other | GI_Hepatic | 0.999209 | 6.57E-76 | 0.945036 | 2.14E-27 |
| Haematological | Respiratory | 0.999189 | 1.26E-75 | 0.929186 | 1.44E-24 |
| Other | Other | 0.999186 | 1.38E-75 | 0.946073 | 1.31E-27 |
| Respiratory | GI_Hepatic | 0.999158 | 3.37E-75 | 0.985181 | 2.90E-42 |

**Table S3. Pairwise disease profile correlations across systems**/ Top-ranking pairwise correlations between disease profiles derived from the 55-analyte time-series feature space. Pearson (linear) and Spearman (rank-based) correlation coefficients with corresponding p-values are shown for representative high-similarity profile pairs across physiological systems.
These results demonstrate near-universal high concordance between disease profiles (Pearson r ≈ 0.999), indicating strong global similarity in analyte patterns across systems, with consistently significant monotonic relationships (Spearman ρ up to ~0.99), supporting a shared underlying structure in the digital blood phenotypic space. The Pearson confidence intervals were also within the 0.99 to 0.999 range.

**Figure S1. Top 20 time-series feature effect sizes (Hedges’ g): Haematological vs. Rest**
Top 20 discriminative longitudinal features ranked by Hedges’ g comparing haematological disease profiles to all other systems. Effect sizes are consistently negative, reflecting reduced values in haematological conditions across repeated timepoints (t2–t5). Dominant signals arise from erythroid (RBC), leukocyte (segmented neutrophils), and red cell morphology (MCV) indices, demonstrating stable, time-resolved separation of haematological profiles.

**Feature Importance Analysis using Machine Learning Multiclass Validation**

The analysis identified a consistent set of highly robust and discriminative features, with longitudinal diagnostic and monitoring signatures (e.g., *fbc_dx_longitudinal_with_signature*, *fbc_dx_single_with_signature*, and *monitor_longitudinal_without_signature*) ranking highest across all importance metrics and exhibiting near-perfect top-feature frequency (≈1.0). These features also demonstrated the lowest mean ranks and minimal variability, indicating strong stability across bootstrap resampling. In contrast, system-level categorical features (e.g., cardiovascular, neuro, thrombosis) showed minimal importance and low selection frequency, suggesting that classification performance is driven primarily by structured hematologic signatures rather than broad disease labels. The convergence of Gini, permutation, SHAP, and bootstrap metrics further confirms that feature importance is robust and not ML method dependent. Collectively, these results support a stable pattern, i.e., a reproducible feature hierarchy underlying disease separability.

**Table S4. Integrated feature importance and robustness analysis across machine learning and statistical validation methods.** Feature importance was evaluated across multiple complementary methods, including Random Forest Gini importance, permutation importance (mean ± SD), SHAP mean absolute values, and bootstrap stability of Gini importance (mean ± SD). Features were further ranked using a consensus mean rank and ranking variability (SD), and their frequency of appearance in the top 20 features across bootstrap iterations was recorded. This integrated framework enables assessment of both discriminative power and robustness of feature contributions across models and resampling strategies.

| seed | procrustes_similarity_to_seed0 | distance_matrix_corr_to_seed0 |
| --- | --- | --- |
| 1 | 0.772276 | 0.906219 |
| 2 | 0.950362 | 0.976061 |
| 3 | 0.96088 | 0.988202 |
| 4 | 0.633593 | 0.916613 |
| 5 | 0.97719 | 0.982387 |
| 6 | 0.960889 | 0.977868 |
| 7 | 0.822286 | 0.920419 |
| 8 | 0.705172 | 0.942354 |
| 9 | 0.775079 | 0.947069 |
| 10 | 0.852676 | 0.97287 |
| 11 | 0.932431 | 0.978718 |
| 12 | 0.83072 | 0.940835 |
| 13 | 0.966421 | 0.97497 |
| 14 | 0.915191 | 0.954964 |
| 15 | 0.93139 | 0.954894 |
| 16 | 0.832076 | 0.97501 |
| 17 | 0.95007 | 0.978638 |
| 18 | 0.89869 | 0.97217 |
| 19 | 0.757409 | 0.959571 |

**Table S5. UMAP Embedding Stability Across Random Seeds (Global Feature Space)**
Assessment of embedding stability across multiple random initializations (seeds 1–19) relative to a reference embedding (seed 0), computed on the global aggregated feature space derived from profile-level representations (machine learning/AID feature set) rather than time-series analytes. Stability was quantified using Procrustes similarity of UMAP embeddings and correlation of pairwise distance matrices.

High Procrustes similarity and distance matrix correlations indicate preservation of global and local structure across stochastic embedding runs. UMAP embeddings demonstrated high reproducibility across seeds, with Procrustes similarity frequently exceeding 0.90 and distance correlations consistently high (≈0.94–0.99). Despite minor variability in layout (expected for stochastic methods), the underlying relational structure between disease profiles remained stable. These results confirm that the learned disease representation in the global feature space is robust to stochastic embedding variation.

| **seed** | **procrustes_similarity_vs_seed0** | **distance_matrix_corr_vs_seed0** |
| --- | --- | --- |
| 1 | 0.793096 | 0.991479 |
| 2 | 0.948366 | 0.996104 |
| 3 | 0.968621 | 0.99368 |
| 4 | 0.991399 | 0.997421 |
| 5 | 0.952584 | 0.996354 |
| 6 | 0.872759 | 0.941837 |
| 7 | 0.750205 | 0.984684 |
| 8 | 0.989389 | 0.998995 |
| 9 | 0.991015 | 0.997774 |
| 10 | 0.976072 | 0.99818 |
| 11 | 0.991138 | 0.997539 |
| 12 | 0.986424 | 0.998606 |
| 13 | 0.860971 | 0.984069 |
| 14 | 0.912811 | 0.995184 |
| 15 | 0.477368 | 0.982724 |
| 16 | 0.980256 | 0.997966 |
| 17 | 0.971898 | 0.993647 |
| 18 | 0.988232 | 0.998948 |
| 19 | 0.824567 | 0.94936 |

**Table S6. UMAP Embedding Stability Across Random Seeds (Time-Series Feature Space)**

Assessment of embedding stability across multiple random initializations (seeds 1–19) relative to a reference embedding (seed 0), computed on the 55-feature longitudinal time-series space. Stability was quantified using Procrustes similarity of UMAP embeddings and correlation of pairwise distance matrices.

High distance matrix correlations (≈0.94–0.999) indicate strong preservation of global pairwise relationships across stochastic embedding runs, while Procrustes similarity shows moderate-to-high variability (≈0.48–0.99), reflecting sensitivity of absolute layout to initialization. Despite this variability, the underlying relational structure between profiles remains consistent.

These results indicate that the time-series feature space exhibits stable global structure, but greater geometric variability compared to the learned global feature space, as dynamical systems, consistent with a lower-dimensional and less constrained representation.

**Figure S2. UMAP stability and noise sensitivity in the 55-time-series feature space.**
(A–C) UMAP embeddings of the 55-feature longitudinal time-series space across different random initializations (seeds 2, 9, and 19) demonstrate consistent global organization despite stochastic variation in layout. (D) Procrustes similarity across seeds confirms high embedding reproducibility, indicating preservation of pairwise relationships and overall structure.
(E–F) UMAP embeddings under increasing Gaussian noise (σ = 0.01 and σ = 0.1) show that the dominant structural pattern remains stable under low-to-moderate perturbations, with only minor deformation at higher noise levels. Together, these results indicate that the time-series feature space captures a robust but low-dimensional structure, where global relationships are preserved across both stochastic initialization and noise perturbation.

**AID Perturbation Analysis**

Algorithmic Information Dynamics (AID) is a causal inference method in silico which combines algorithmic information theory and graph perturbation analysis to investigate the dynamics and complexity of complex networks/systems. AID asks how much the algorithmic information content of an underlying topology or representation changes when a network feature (i.e., an analyte, node, or edge) is perturbed in the disease-feature matrix. Features with the largest perturbation shifts among its complexity metrics are interpreted as the most structurally and causally influential. Thus, algorithmic information signatures, using Block Decomposition Method (BDM) perturbation, serve as predictors and markers of the underlying generative structure of a complex system/process. BDM estimates a graph’s Kolmogorov complexity—the length of the shortest computer program capable of generating that network.

In our data, the strongest and most recurrent shifts are concentrated in a small group of features, especially screening- and diagnosis-related longitudinal signatures and selected FBC-derived signatures. This further validates our findings that a limited set of features appears to carry disproportionate causal or control-like influence over the clustering pattern (representation learning). Thus, AID perturbation analysis and model-agnostic robustness complements the supervised network physiology and feature-importance results in the Main Text by highlighting structurally sensitive features across multiple complexity measures. Each feature was binarized across multiple thresholds (t = 0.2, 0.35, 0.5, 0.65, 0.8), and perturbation shifts were quantified using various complexity metrics as shown in Figure S3.

**Figure S3 Top. Algorithmic Information Dynamics (AID) perturbation analysis of feature sensitivity**. (A) Top features ranked by mean absolute BDM perturbation shift. (B) Threshold-dependent BDM perturbation profiles across binarization levels. (C) Cross-measure comparison of perturbation sensitivity using BDM, entropy, and compression-based metrics. These analyses identify structurally influential features within the hematologic feature space. **Bottom. BDM Shift Matrix Across Thresholds for Full Time-Series Disease-Feature Matrix.** Heatmap of BDM compelxity shifts for all analytes across a range of binarization thresholds (0.2–0.8) in the 55-feature time-series disease matrix. A matrix is a 2D representation of a graph network. Rows represent longitudinal analytes (e.g., WBC, haemoglobin, RBC, neutrophils), and columns represent threshold levels used in AID perturbation analysis. Higher BDM shifts indicate greater structural influence on the global information landscape. The matrix reveals consistent, high-magnitude perturbations in key hematologic features across multiple timepoints, supporting their role as dominant contributors to the system’s underlying attractor-like structure
